# A genetic pathway for naturally colored cotton production

**DOI:** 10.1093/plphys/kiac168

**Published:** 2022-04-13

**Authors:** Lijuan Zhou, Yajin Ye

**Affiliations:** Key Laboratory of Forest Genetics and Biotechnology of Ministry of Education of China, Co-Innovation Center for the Sustainable Forestry in Southern China, Nanjing Forestry University, Nanjing 210037, China; Key Laboratory of Forest Genetics and Biotechnology of Ministry of Education of China, Co-Innovation Center for the Sustainable Forestry in Southern China, Nanjing Forestry University, Nanjing 210037, China

Naturally colored cotton (NCC) results from pigmentation in developing fibers. Importantly, NCC serves as an environmental-friendly resource for human society because it does not require dyeing and bleaching during textile processing, and thus substantially reduces pollution and saves energy and water (Wen et al., 2018; Wang et al., 2022b). With the increasing demand for eco-friendly products, NCCs have attracted more interest from the textile industry. Currently, NCC yields are lower and the NCC fiber is usually shorter and weaker than regular cotton, limiting NCC’s applications. There is an urgent need to understand the genetic basis of the natural brown cotton to improve the breeding of this green product (Yan et al., 2018).

Brown cotton fibers are the most frequently used NCCs, and the coloration is the result of excessive accumulation of anthocyanin and proanthocyanidin (PA). Biosynthesis, transport, and storage pathways of these pigments have been well characterized in Arabidopsis (*Arabidopsis thaliana*) (Martin et al., 1991; Holton and Cornish, 1995). However, only a few genes related to anthocyanin homeostasis have been cloned and functionally characterized in cotton (*Gossypium hirsutum*) (Wen et al., 2018; Yan et al., 2018). In this issue of *Plant Physiology*, Wang and colleagues report that the transcription factor MYB113 regulates anthocyanin biosynthesis in cotton. Ectopic expression of *MYB113* activated anthocyanin and PA synthesis, resulting in red foliated cotton and brown fiber (Wang et al., 2022a).

The authors isolated a red foliated cotton mutant (designated as *Re*S11) from the backcross progenies between sea-island cotton and upland cotton. Map-based cloning identified a 288-bp insertion in the promoter region of *MYB113* in *Re*S11. The insertion contains a G-box, which caused increased expression of *MYB113* in the mutant. The authors further demonstrated that ectopic overexpression of *MYB113* under the control of the *Cauliflower Mosaic Virus 3*5S promoter caused the red foliated cotton phenotype. The *35S:MYB113* overexpression transgenic plants developed red color in various organs, but the fiber remained white. Interestingly, fiber-specific over-expression of *MYB113* resulted in brown mature fiber, and the color change was caused by overaccumulation of anthocyanin and PA. In addition, anthocyanin biosynthetic genes were highly induced in the *MYB113* overexpression lines, which further established the direct connection between anthocyanin accumulation and brown fibers.

The authors conducted transcriptome analysis and co-expression network analysis to identify genes in the same genetic pathways as that of *MYB113*. As a result, a transcriptional regulatory network of *MYB113* for the anthocyanin pathway was proposed and further validated by the dual-luciferase reporter system. In this network, phytochrome interacting factor 4 (PIF4) binds to the G-box region of the *MYB113* promoter and drives the expression of *MYB113*, which transcriptionally activates genes in anthocyanin biosynthesis and metabolism, leading to increased anthocyanin synthesis. Consequently, the *Re* mutant with the G-box-containing insertion produces red foliated cotton (Figure 1).

**Figure 1:**
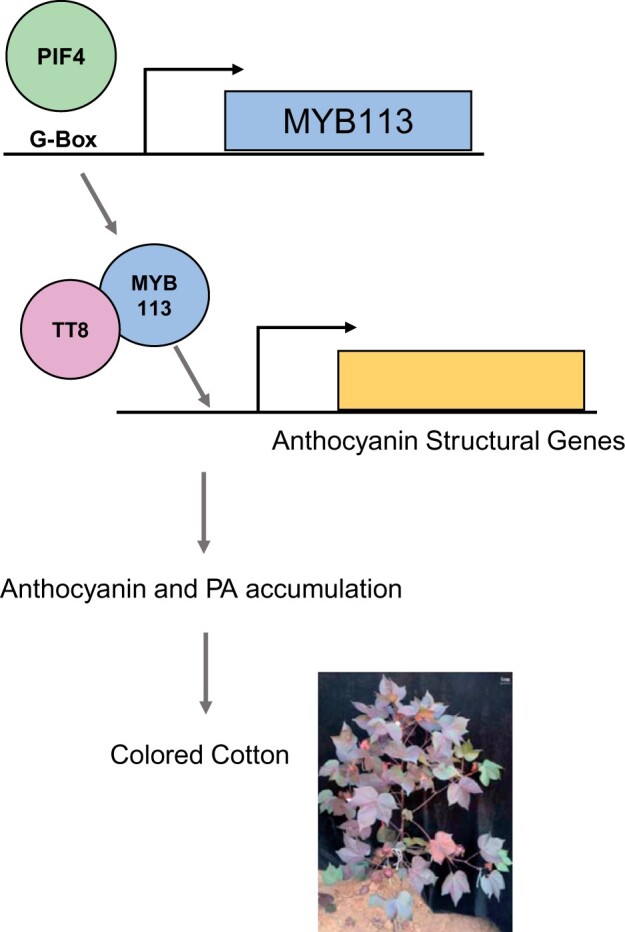
The regulatory model controlling brown coloration by the MYB113 transcription factor. Light-responsive factor PIF4 binds to the G-Box region in the promoter of *MYB113* to drive its expression. Afterward, MYB113 forms a protein complex with TT8 and activates the expression of anthocyanin biosynthesis genes, leading to the accumulation of anthocyanin and PA. The increased pigmentation produces red foliated cotton and brown fiber. Coding genes are depicted as bars with line and arrow showing promoter and transcription direction, respectively. Proteins are represented with circles.

The MYB113-anthocyanin module characterized by Wang et al. (2022a) offers an excellent strategy for molecular breeding of NCC. In-depth understanding of how other genetic factors associated with MYB113 function in this module will be crucial for expanding the molecular targets for colored cotton fiber breeding practices (Gonzalez et al., 2008). Another challenge ahead is to precisely control the expression of color-producing genes to find the balance between coloration and ﬁber quality and/or productivity, and thus reach the best economic benefit.

*Conflict of interest statement*. None declared.

## References

[kiac168-B1] Gonzalez A , Zhao M, John ML, Alan ML (2008) Regulation of the anthocyanin biosynthetic pathway by the TTG1/bHLH/Myb transcriptional complex in Arabidopsis seedlings. Plant J 53: 814–827 18036197 10.1111/j.1365-313X.2007.03373.x

[kiac168-B2] Holton TA , Cornish EC (1995) Genetics and biochemistry of anthocyanin biosynthesis. Plant Cell 7: 1071–1083 12242398 10.1105/tpc.7.7.1071 PMC160913

[kiac168-B4] Martin C , Prescott A, Mackay S, Bartlett J, Vrijlandt E (1991) Control of anthocyanin biosynthesis in flowers of *Antirrhinum majus*. Plant J 1: 37–49 1844879 10.1111/j.1365-313x.1991.00037.x

[kiac168-B5] Wang N , Zhang B, Yao T, Shen C, Wen T, Zhang R, Li Y, Le Y, Li Z, Zhang X, et al (2022a) Re enhances anthocyanin and proanthocyanidin accumulation to produce red foliated cotton and brown fiber. Plant Physiol **189**: 1466–1481 10.1093/plphys/kiac118 PMC9237731 35289870

[kiac168-B6] Wang Z , Zhang X, He S, Rehman A, Jia Y, Li H, Pan Z, Geng X, Gao Q, Wang L, et al (2022b) Transcriptome co-expression network and metabolome analysis identifies key genes and regulators of proanthocyanidins biosynthesis in brown cotton. Front Plant Sci 12: 822198 35237281 10.3389/fpls.2021.822198 PMC8882990

[kiac168-B7] Wen T , Wu M, Shen C, Gao B, Zhu D, Zhang X, You C, Lin Z (2018) Linkage and association mapping reveals the genetic basis of brown fiber (*Gossypium hirsutum*). Plant Biotechnol J 16: 1654–1666 10.1111/pbi.12902 PMC6097129 29476651

[kiac168-B8] Yan Q , Wang Y, Li Q, Zhang Z, Ding H, Zhang Y, Liu H, Luo M, Liu D, Song W, et al (2018) Up-regulation of GhTT2-3A in cotton fibres during secondary wall thickening results in brown fibers with improved quality. Plant Biotechnol J 16: 1735–1747 29509985 10.1111/pbi.12910 PMC6131414

